# Fingerprinting Soybean Germplasm and Its Utility in Genomic Research

**DOI:** 10.1534/g3.115.019000

**Published:** 2015-07-28

**Authors:** Qijian Song, David L. Hyten, Gaofeng Jia, Charles V. Quigley, Edward W. Fickus, Randall L. Nelson, Perry B. Cregan

**Affiliations:** * United States Department of Agriculture, Agricultural Research Service, Soybean Genomics and Improvement Laboratory, Beltsville, Maryland 20705-2350; † Pioneer Hi-Bred International Inc, Johnston Iowa 50131-0184; ‡ United States Department of Agriculture, Agricultural Research Service, Soybean/Maize Germplasm, Pathology and Genetics Research Unit and Department of Crop Sciences, University of Illinois, Urbana, Illinois 61801-0000

**Keywords:** soybean, germplasm, genotyping, SoySNP50K, genetic diversity, haplotype block map

## Abstract

The United States Department of Agriculture, Soybean Germplasm Collection includes 18,480 domesticated soybean and 1168 wild soybean accessions introduced from 84 countries or developed in the United States. This collection was genotyped with the SoySNP50K BeadChip containing greater than 50K single-nucleotide polymorphisms. Redundant accessions were identified in the collection, and distinct genetic backgrounds of soybean from different geographic origins were observed that could be a unique resource for soybean genetic improvement. We detected a dramatic reduction of genetic diversity based on linkage disequilibrium and haplotype structure analyses of the wild, landrace, and North American cultivar populations and identified candidate regions associated with domestication and selection imposed by North American breeding. We constructed the first soybean haplotype block maps in the wild, landrace, and North American cultivar populations and observed that most recombination events occurred in the regions between haplotype blocks. These haplotype maps are crucial for association mapping aimed at the identification of genes controlling traits of economic importance. A case-control association test delimited potential genomic regions along seven chromosomes that most likely contain genes controlling seed weight in domesticated soybean. The resulting dataset will facilitate germplasm utilization, identification of genes controlling important traits, and will accelerate the creation of soybean varieties with improved seed yield and quality.

Domesticated in China, the cultivated soybean [*Glycine max* (L). Merr.] was first introduced in North America in 1765 (Hymowitz and Harlan 1983). Extensive soybean collecting started in the 1920s but systematic preservation did occur until the United States Department of Agriculture (USDA) Soybean Collection was established in 1949 (Carter *et al.* 2004). Few wild soybean [*Glycine soja* (Sieb. and Zucc.)] accessions were added to the collection until the 1970s. At the time this project was initiated, the USDA Soybean Germplasm Collection contained approximately 19,700 soybean accessions. The collection includes more than 1100 wild soybeans from China, Korea, Japan and Russia, and more than 18,000 cultivated soybeans from China, Korea, Japan, and 84 other countries. Most of the cultivated soybean from China, Korea, and Japan are landraces that are not the product of modern plant breeding. The earliest soybean accession in the USDA Soybean Germplasm Collection was collected before 1895. The accessions in the Collection are the sources of genes for soybean improvement not just in the North America (N. Am.), but worldwide.

Although some of the accessions in the USDA Soybean Germplasm Collection have been evaluated for their biotic and abiotic stress resistance, seed constituents and productivity during the past decades, knowledge of molecular variation, diversity, genetic architecture, and selection that may underlie the phenotypic variation in the soybean genome based on the entire soybean collection is unknown. Previous studies have documented the existence of linkage disequilibrium (LD) (Hyten *et al.* 2007; Lam *et al.* 2010; Li *et al.* 2008; Zhu *et al.* 2003), genomic regions of selection (Lam *et al.* 2010), and the level of genetic diversity (Hyten *et al.* 2006; Lam *et al.* 2010; Li *et al.* 2008; Zhu *et al.* 2003) among various soybean populations. However, these studies were of limited scale in terms of sample size and/or the number of loci analyzed.

Highly selfing species, like soybean, are in many ways uniquely suitable for haplotype block mapping. Domestication and artificial selection have led to extensive LD and haplotype structure. The availability of inbred accessions makes it possible to observe rather than infer haplotypes. Whole-genome haplotype block mapping has been proposed in humans as a powerful tool to detect genes conditioning complex traits by limiting the number of single-nucleotide polymorphisms (SNPs) to be typed (Cardon and Abecasis 2003; Chien *et al.* 2013; Daly *et al.* 2001; Gabriel *et al.* 2002; Zhang *et al.* 2004a).

The N. Am. soybean crop accounts for 29% of world production; however, it may be at a low level of diversity due to several genetic bottlenecks. These include the domestication from wild soybean, the limited set of introduced landraces that form the N. Am. soybean genetic base, and intensive selection for enhanced agronomic performance (Hyten *et al.* 2006). Determination of the genomic regions affected by, and the extent of fixation and LD caused by the domestication, introduction, and selection bottlenecks on a large population scale are fundamental information related to the future genetic improvement of soybean.

Here we report the molecular analysis of the soybean and wild soybean accessions in the USDA Soybean Germplasm Collection with a high-density genotyping array of soybean, the SoySNP50K beadchip (Song *et al.* 2013), which has been the largest such analysis in a plant species to date. The fingerprinting of the USDA Soybean Germplasm Collection provided a definition of soybean LD and haplotype block structure across the genome, identified genomic regions associated with soybean domestication and selection in N. Am. breeding programs, and delimited regions associated with seed weight, an important component of seed yield.

## Materials and Methods

### Soybean germplasm and DNA analysis

The seeds of 19,648 accessions including 1168 wild and 18,480 cultivated soybean accessions in the USDA Soybean Germplasm Collection (Urbana, IL) were crushed to a powder in a deep-well microtiter plate (Thermo Scientific AB-0932) with a steel ball using a Retsch MM400 Mixer Mill at 30/s for 2 min, and DNA was extracted using the cetyltrimethylammonium bromide method (Keim *et al.* 1988).

### SNP genotyping

A high-throughput SNP assay, the SoySNP50K Illumina Infinium II BeadChip containing 52,041 SNPs that were chosen from euchromatic and heterochromatic genome regions, was used for genotyping. SNP genotyping was conducted following the procedures described previously (Song *et al.* 2013). Any polymorphic SNP with a rate of missing and heterozygous allele calls greater than 0.1 among the 19,648 soybean and wild soybean accessions was eliminated. The heterozygous allele calls in the remaining loci were set as missing in the subsequent analysis. The position of SNPs in the soybean genome was based on the Glyma1.01 assembly.

### Similarity analysis

Genetic similarity between pairs of genotypes among the 18,480 cultivated and among the 1168 wild accessions was calculated as the ratio of the number of identical SNP allele calls and the total number of SNPs for which allele calls were made for the pair.

### Cluster analysis

Pair-wise distance among the accessions of 806 wild and 5396 landrace soybeans was obtained based on the allelic dissimilarity of the 42,509 SNPs; the neighbor-joining tree was constructed using the software Mega 5.10 (Tamura *et al.* 2011).

### LD analysis

LD was analyzed within the wild, landrace, and N. Am. cultivar populations with 806, 5396, and 562 accessions, respectively. Only the SNPs with minor allele frequency ≥5% were included for LD calculation and construction of haplotype blocks. Calculation of pairwise LD (*r*^2^) among SNPs and identification of haplotype blocks was based upon SNPs within 1-Mb windows using the software PLINK (Purcell *et al.* 2007). Haplotype blocks were identified through estimates of *D*′ for all pairwise combinations of SNPs. Pairs of SNPs were in a haplotype block if the one-tailed upper 95% confidence limit on D′ was greater than 0.98 and the lower limit was greater than 0.7 (Gabriel *et al.* 2002).

### Haplotype block sharing

To compare block boundaries among wild soybeans, landraces, and N. Am. cultivars populations, the ratio of haplotype sharing across populations was calculated based on the method of Gabriel *et al.* (2002) with modification. SNPs in the haplotype blocks of the two populations being compared were identified and concordance of all SNP pairs in the same block of one population *vs.* SNP pairs in the other population was examined. A SNP pair was concordant if the pair was assigned to the same block in both populations, and non-concordant if the assignment was not to the same block. The percentage of haplotype block sharing was calculated as: [the number of concordant SNPs/(number of concordant SNPs + number of nonconcordant SNPs)] × 100.

### Calculation of fixation index (F_st_)

The F_st_ between the wild and landrace genotypes and the F_st_ between the landrace and N. Am. cultivars were calculated using the software Arlequin v3.1 (Excoffier *et al.* 2005).

### Expected number of genes in and between haplotype blocks

The expected number of genes in and between the haplotype blocks of the euchromatic and heterochromatic regions of each population was calculated on the basis of an even distribution of genes per physical distance in both the euchromatic and heterochromatic regions. The positions of the 46,430 high-confidence protein coding genes were downloaded at http://www.phytozome.net, and the observed number of genes in and between the blocks of the euchromatic and heterochromatic regions of each population was counted.

### Recombination rate in and between haplotype blocks

The recombination rate in centimorgans within haplotype blocks was estimated based upon the genetic distance of 21,483 SNPs in the linkage map developed via the analysis of a Williams 82 × PI479752 population with 1083 recombinant inbred lines that were genotyped with the SoySNP50K chip. Total genetic distance, as well as total length of sequence (bp) between the most distant SNPs in haplotype blocks, was calculated to estimate the observed and expected recombination rate within haplotype block regions.

### Genome-wide association analysis of seed weight

For the purpose of genome wide association analysis of seed weight (g/100 seeds), the dataset containing seed weight of the *G. max* accessions was downloaded from the Germplasm Resources Information Network (GRIN), http://www.ars-grin.gov/npgs/searchgrin.html. Given that the seed weight of the accessions was observed in different years and/or environments, variation of seed weight impacted by different growing environments was expected. To eliminate such variation, we used a method to treat seed weight as a dichotomous, *i.e.*, 0, and 1, rather than a quantitative trait. If the seed weight is ≥20 g/100 seeds, the seed weight was set to 1, and if the seed weight is ≤10 g/100 seeds, the value was set to 0. Thus, only 3753 accessions with seed weight ≥20 g or ≤10 grams per 100 seeds were included in the association analysis. It is very unlikely that the seed weight classification of the accessions based on this standard would be affected by environmental effects among years and locations. Then, a genome-wide association study of the seed size was executed with the PLINK procedure “case-control association test,” taking into account population stratification with a defined number of clusters, K. The K estimation of the population was obtained using the software fastStructure (Raj *et al.* 2014), K = 2−15 was chosen. A value of –log(p), where the p is the genomic control adjusted significance level of each locus was obtained from PLINK. Significant loci were identified when the –log(p) value was greater than 3.

### Data availability

The dataset for 19,648 soybean Plant Introductions genotyped with the SoySNP50K BeadChip is available at SoyBase, the USDA-ARS Soybean Genetics and Genomics Database http://soybase.org/snps/download.php).

## Results

### Redundant and highly similar soybean accessions

Of the 52,041 SNPs in the SoySNP50K bead pool, a total of 42,509 SNPs were polymorphic and had a rate of missing and heterozygous allele calls <0.1 among the 19,648 soybean and wild soybean accessions characterized. Based on the pair-wise genetic similarity of the accessions calculated from the 42,509 SNPs, 1682 and 95 accessions were 100% identical to at least one other accession among the 18,480 *G. max* and 1168 *G. soja* accessions, respectively. Overall, 4303 *G. max* (23%) and 362 *G. soja* (30%) accessions were at least 99.9% identical to another accession in the collection (Table 1).

**Table 1:** Number of accessions in the USDA Soybean Germplasm Collection with similarity >99.9% based on SNP comparisons

Similarity Among Accessions	*G. soja*	*G. max*
Accessions 100% similar to another accession	95	1682
Accessions >99.9% similar to another accession	362	4306
Proportion of identical accessions (%)	(95/1168) = 8%	(1682/18480) = 9%
Proportion of accessions with >99.9% similar (%)	(362/1168) = 30%	(4306/18480) = 23%

USDA, United States Department of Agriculture; SNP, single-nucleotide polymorphism.

After eliminating all but one accession within each group of accessions with greater than 99.9% similarity, a total of 806 *G. soja* accessions from China, Korea, Japan, and Russia and 14,181 *G. max* accessions remained. Of the 14,181 *G. max* accessions, a total of 5396 landraces from China, Korea, and Japan and 562 N. Am. cultivars were used for further analysis (Supporting Information, Table S1).

### Genetic relationship with geographic origins of soybean accessions

**Figure 1:**
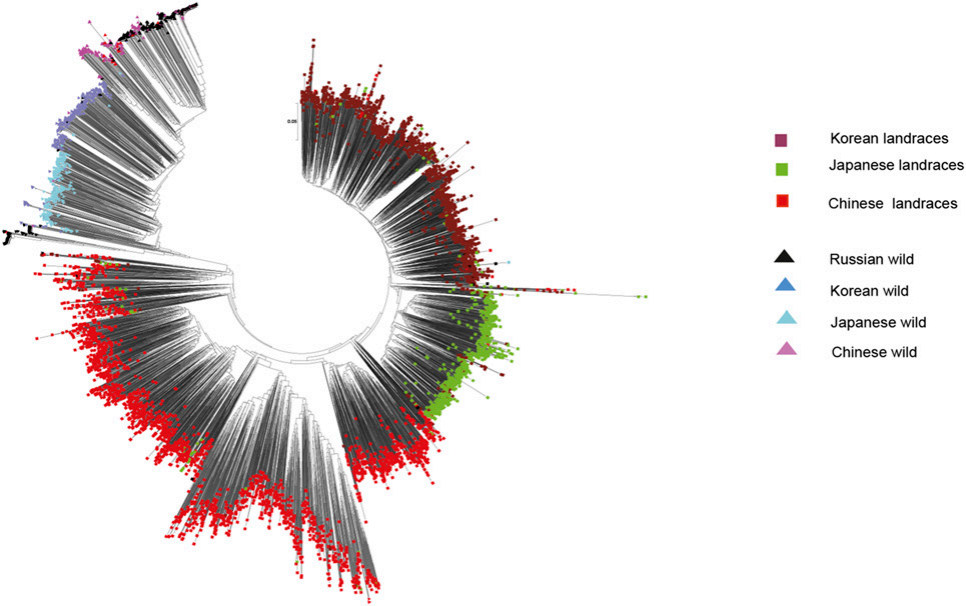
Dendrogam of wild and landrace genotypes from different countries.

Cluster analysis of the 806 wild soybean accessions from China, Korea, Japan, and Russia and 5396 landraces from China, Korea, and Japan showed that wild soybeans and landraces from different countries were well separated and the clusters of wild soybean and soybean landraces were consistent with their geographic origins (Figure 1). This indicates that landraces from China, Korean, and Japan have distinct genetic backgrounds that are unique and that could be useful in genetic soybean improvement.

### Extent of LD among wild, landrace, and N. Am. cultivar populations

**Figure 2:**
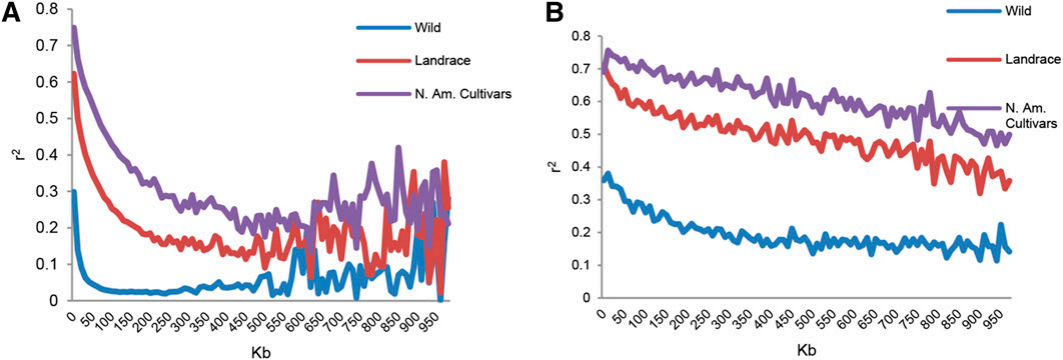
Linkage disequilibrium (LD) in euchromatic and heterochromatic regions. (A) LD in euchromatic regions of wild, landrace, and North American (N. Am.) cultivar soybean populations. (B) LD in heterochromatic regions of the wild, landrace, and N. Am. cultivar soybean populations.

The trend of LD decay among the 806 wild, 5396 landrace, and 562 N. Am. cultivar populations was very similar in the euchromatic and heterochromatic regions. The LD was more extensive in the N. Am. cultivars than in the landraces, and in the landraces than in the wild soybeans. LD was much more extensive in the heterochromatic than in the euchromatic regions in each population (Figure 2). In the euchromatic regions, the LD declined to half of its maximum value within approximately 20 kb, 75 kb, and 160kb in the wild, landrace, and N. Am. cultivar populations, respectively, whereas in the heterochromatic regions, the LD declined to half of its maximum value within 350 kb, 900 kb, and 970 kb in the wild, landrace, and N. Am. cultivar populations, respectively. Thus, in both regions, LD was the lowest in the wild soybean and the highest in the N. Am. cultivars.

### Haplotype map and haplotype block structure among wild, landrace, and N. Am. cultivar populations

The total number of haplotype blocks ranged from 3000 to 5300 among the 806 wild, 5396 landrace, and 562 N. Am. cultivar populations (Table S2, Table S3, and Table S4). In each of the populations, most of these blocks resided in the euchromatic regions where there were 4331 blocks in the wild, 4777 in the landrace, and 2763 in the N. Am. cultivar population. The average block size was 10.7, 39.6, and 79.6 kb, respectively, in the three populations (Table 2). Similarly, in the heterochromatic regions, the smallest average block size was detected in the wild and the largest in the N. Am. cultivar population. However, the average haplotype block size was much smaller in euchromatic regions *vs.* the heterochromatic regions in all three populations. Approximately 10%, 41%, and 48% of the euchromatic regions were in haplotype blocks in the wild, landrace, and N. Am. cultivars, respectively. The percentage of the heterochromatic regions in haplotype blocks was also the lowest in the wild, followed by the N. Am. cultivar and landrace populations. Most of the haplotype blocks (>83.7%) were shared between any two of the three populations (Table S5).

**Table 2:** Number of haplotype blocks and their size in the wild, landrace, and N. Am. cultivar soybean populations

Population	Genome-Wide			Euchromatic Regions				Heterochromatic Regions			
	Number of Accessions	Number of SNPs in Blocks	Total Blocks	Number of Blocks	Total Sequence Length in Blocks, kb	Average Block Size, kb	Proportion of Euchromatic Regions in Blocks	Number of Blocks	Total Sequence Length in Blocks, kb	Average Block Size, kb	Proportion of Heterochromatic Regions in Blocks
Wild	806	14,343	4624	4331	46,246	10.7	0.10	293	149,958	511.8	0.31
Landrace	5396	28,111	5226	4777	189,134	39.6	0.41	449	281,061	626.0	0.57
N. Am. cultivars	562	24,753	3093	2763	219,872	79.6	0.48	330	222,619	674.6	0.45

N. Am., North American; SNPs, single-nucleotide polymorphisms.

**Figure 3:**
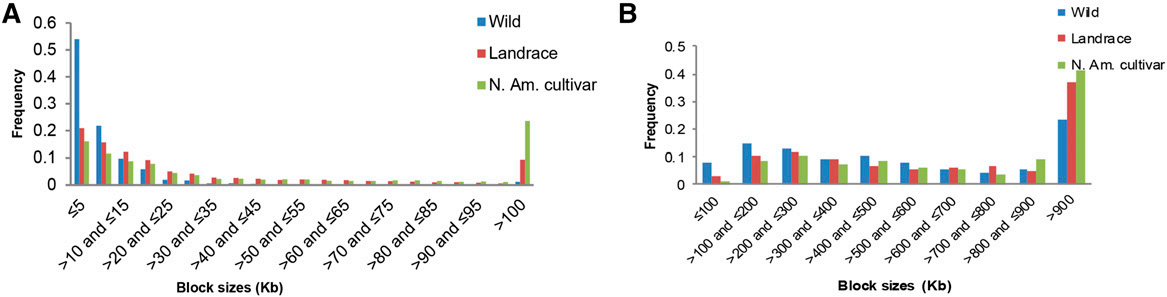
Distribution of haplotype block size. (A) Haplotype block size in euchromatic regions of wild, landrace, and North American (N. Am.) cultivar populations. (B) Haplotype block size in heterochromatic regions of the wild, landrace, and N. Am. cultivar populations.

Analysis of the distribution of haplotype block size in euchromatic regions showed that although >50% of the haplotype blocks extended <15 kb in the landrace and <20 kb in the N. Am. cultivar accessions, there were 10% and 24% of the blocks with size >100 kb in the landrace and N. Am. cultivar accessions, respectively (Figure 3A). In the wild soybean, more than 50% of the blocks were less than 5 kb and less than 1% of the blocks were greater than 100 kb. In the heterochromatic regions, a large proportion of the blocks were >900 kb in size, which included 42% of the blocks in the N. Am. cultivar accessions followed by 37% in the landraces and 24% in the wild soybean population (Figure 3B). Clearly, the wild soybean haplotype block structure is characterized by much smaller blocks. In terms of haplotype diversity, the three populations were quite similar with the mean number of haplotypes/block of 3.4, 3.5, and 3.7 in the wild, N. Am. Cultivar, and landrace populations, respectively (Table 3). Among these, >66% of the haplotypes in euchromatic and >55% in heterochromatic regions had a frequency >10% among the three populations (Table S6). The relatively low number of haplotypes/block was surprising, given the fact that the average number of SNPs per block ranged from 2.9 to 7.8 in the euchromatic regions and from 6.8 to 10.2 in the heterochromatic regions of the wild, landrace and the N. Am. cultivar populations.

**Table 3:** Haplotype block structure in the wild, landrace, and N. Am. cultivar soybean populations

Population	Genome-Wide		Euchromatic Regions				Heterochromatic Regions			
	Number of Haplotypes	Number of Haplotypes/ Block	Number of Haplotypes	Number of Haplotypes/Block	Number of SNPs in Blocks	Number of SNPs/Block	Number of Haplotypes	Number of Haplotypes/Block	Number of SNPs in Blocks	Number of SNPs/Block
Wild	15,890	3.4	14,356	3.3	12,417	2.9	1534	5.2	1984	6.8
Landrace	19,529	3.7	17,617	3.7	23,990	5.0	1912	4.3	4121	9.2
N. Am.	10,933	3.5	9753	3.5	21,435	7.8	1180	3.6	3361	10.2

N. Am., North American; SNPs, single-nucleotide polymorphisms.

Although the number of genes in haplotype blocks was slightly lower than expectation in the heterochromatic regions of the three populations and higher than expectation in the euchromatic regions of the landrace and N. Am. cultivar populations, the bias of gene density within blocks *vs.* between blocks was not severe (Table S7). However, as expected, the recombination rate within blocks was dramatically reduced and was approximately only 12–39% and 12–41% of expectation in the blocks of the euchromatic and heterochromatic regions, respectively, in the wild, landrace, and N. Am. cultivar populations (Table S8). The wild population had the most dramatic reduction in recombination frequency and the N. Am. cultivar population had the least.

### Genomic regions associated with domestication and selective sweeps associated with soybean breeding

Genome-wide F_st_ was 0.23 between the wild soybean (806 accessions) and landrace (5396 accessions) populations (Table S9) with a standard deviation of 0.242 and 0.11 between the landrace and N. Am. cultivar (562 accessions) populations, with a standard deviation of 0.147. Thus, the threshold of F_st_ with a two-tailed significance level of 5% was 0.704 and 0.398 between the wild and landrace and between the landrace and N. Am. cultivar populations, respectively. Analysis of average F_st_ and the distribution of individual loci above the F_st_ threshold at the 5% significance level among chromosomes suggested that chromosomes underwent different selective pressure during domestication *vs.* that imposed by modern breeding. For example, the average F_st_ between the wild and landrace populations was much greater along Gm12 (0.315) than Gm18 (0.174), and there was a much greater proportion of loci with significant F_st_ (14.05% *vs.* 1.59%) on Gm12 *vs.* Gm18. The F_st_ between the landrace and N. Am. cultivar populations ranged from 0.060 on Gm05 to 0.157 on Gm19. The proportion of loci with F_st_ ≥0.398 was 11.86% on Gm19 *vs.* 2.21% on Gm05 (Table S10).

We examined the potential relationship of the candidate regions with several traits. Determinate *vs.* indeterminate growth habit in the cultivated soybean is thought to be a trait that has undergone strong selection in N. Am. breeding programs. The approximate percentage of accessions with determinate growth habit was 65% in landraces *vs.* 32% in the N. Am. cultivars based on the data from GRIN. The gene Glyma19g37890.1, which is located at 44.93 Mb∼44.97 Mb of Gm19 was identified as the gene conditioning determinacy (Tian *et al.* 2010). In the region, we observed high F_st_ (>0.40) between the landrace and the N. Am. cultivar populations for six of seven SNPs. However, a low F_st_ value was observed between the wild and landrace populations in this region of Gm19. This finding is consistent with the results reported by Tian *et al.* (2010) that determinacy in landraces was the result of a gene mutation and not selection from *G. soja*. A number of SNPs with high F_st_ between the wild and landrace populations were detected in regions previously reported to contain domestication quantitative trait loci (QTL). These included the region at 4.00−7.00 Mb of Gm05 containing a QTL conditioning pod number per plant (Zhang *et al.* 2007), plant height (Chen *et al.* 2007; Kabelka *et al.* 2004), and days to maturity (Kabelka *et al.* 2004) and 2.8−7.1 Mb, 41.1−42.4 Mb, and 46.1−49.1 Mb of Gm19 (Bailey *et al.* 1997; Kang *et al.* 2009) where QTL conditioning pod shattering have been reported.

### Application of genotyping data to the analysis of genome-wide association of seed weight

We identified 1936 and 1817 *G. max* accessions with seed weight ≥20 and ≤10 g/100 seeds in the GRIN database, respectively. FastStructure analysis showed that the appropriate K was 5−15, and the Admixture analysis (Alexander *et al.* 2009) showed that the appropriate K was 12. As the accessions were from 12 maturity groups, the K = 12 was chosen. A total of 30 loci were significantly associated with seed weight and the loci were concentrated in eight regions of chromosomes Gm01 at 32.3 Mb, Gm06 at 19.7 Mb, Gm11 at 28.2−30.0 Mb, Gm13 at 38.4 Mb, Gm14 at 28.5−34.2 Mb, Gm17 at 2.4−2.5 Mb and 8.7−8.8 Mb, and Gm19 at 42.7−43.1 Mb (Figure S1). As shown in Table S11, QTL for seed weight were previously reported in similar genomic regions using recombinant inbred line populations (Gai *et al.* 2007; Hoeck *et al.* 2003; Hyten *et al.* 2004; Mian *et al.* 1996; Panthee *et al.* 2005; Specht *et al.* 2001; Zhang *et al.* 2004b). However, because these were quantitative trait locus analyses of lines derived from single cross populations with the resulting high level of LD, the regions in which the reported seed weight QTL resided were poorly defined and in some cases the reported regions spanned as much as 17 Mb.

The association of the regions with seed size is also consistent with the observation that, with the exception of Gm1:32.3Mb and Gm13:38.4, the remaining regions each contained loci with high Fst (>0.70) between the wild soybean and landrace accessions.

## Discussion

The annual accessions in the USDA Soybean Germplasm Collection were assayed with the SNPs contained on the SoySNP50K (Song *et al.* 2013) BeadChip. The dataset will be instrumental in making large-scale, genome-wide association studies a reality for other laboratories and will help researchers narrow the genomic regions of the targeted QTL. Because the seeds of the soybean germplasm accessions and the DNA genotyping data generated from this study are publically available, researchers will be able to integrate their phenotypic data with the genome-wide SNP data to identify genetic factors that influence their traits of interest, such as biotic and abiotic stress resistance, as well as agronomic and seed quality traits. In addition, this work provides a rich renewable genetic resource for soybean scientists and breeders to study the prevalence of haplotype variation and genetic diversity in soybean germplasm, as well as to identify regions and the specific genes associated with a diversity of traits and tag SNPs for selection of desired genotypes. In a recent report of an association analysis of soybean seed protein and oil content using the set of SNPs described in this report (Hwang *et al.* 2014), 17 genome positions associated with level of seed protein and 13 genome positions associated with seed oil content were identified. A total of 12 of the 17 regions containing protein QTL and eight of the 13 regions with oil QTL had previously been reported based upon QTL analyses. It was concluded that the genome-wide association study not only identified many of the previously reported QTL controlling seed protein and oil, but also resulted in more narrowly defining the genomic regions containing the genes of interest than the regions reported based upon QTL analysis. This was also true for the genome-wide association analysis of seed weight that we report here. These narrower genomic regions will expedite the identification and cloning of the causal genes.

The high rate of redundant and highly similar accessions detected in the USDA Soybean Germplasm Collection is not surprising. Soybean accessions have been acquired from more than 80 countries worldwide and because of incomplete or nonexistent passport information, the same accession with different names have been acquired a number of times. In addition, in countries that are the center of origin of soybean ,such as China, soybean was commonly named by farmers according to soybean seed coat color, seed size and shape, pod color, or the month of maturity, and thus it is very common for the same accession to have different names in different areas of the country. Because a limited number of agronomic or morphological traits are available to distinguish these accessions, profiling each accession in the USDA Soybean Germplasm Collection with a large number of molecular markers is essential to understand the level of repetitiveness, thus increasing the efficiency of germplasm preservation, characterization, and promoting the more efficient utilization of the genetic resources in soybean breeding programs. This research provides the first in-depth analysis of the current soybean germplasm collection in the United States.

The extent of LD and the average haplotype block sizes were the lowest in the wild soybean population, were greater in the landrace population, and were greater still in the N. Am. cultivar population. Studies indicate that long range LD and increased haplotype block size can arise by several different mechanisms, including natural and artificial selection in the form of selective sweeps or background selection and population bottlenecks and founding effects from ancestrally conserved segments (Phillips *et al.* 2003). Domestication is the process of adapting a plant species to the production environment and to the needs of the consumer. This involves changes in the gene pool over generations. During domestication the plant species will differ from its wild counterparts by greatly diminished effective population size, and consequently, increased LD. Modern breeding practices further shrink the effective population size by using a small fraction of accessions from the landrace gene pool such as occurred when the soybean was introduced into North America, *e.g.*, Gizlice *et al.* (1994) identified a group of 14 genotypes which had contributed 80.5% of the allelic diversity present in N. Am. soybean cultivars released between 1947 and 1988 (Gizlice *et al.* 1994).

Subsequently, the extensive use of a small number of elite genotypes in breeding programs further reduces genetic variability. Thus, the extended regions of LD and increased haplotype block size are an indicator of reduced genomic diversity from wild to landrace populations and from landrace to the N. Am. cultivar populations, which was consistent with the previous report that genetic diversity of the landrace and cultivar populations was dramatically reduced by domestication and selection (Hyten *et al.* 2006), respectively.

We have provided the first high-resolution haplotype maps based on the largest sample size and the largest number of loci reported in soybean thus far. With the aid of the haplotype block map, we can efficiently select and tag SNPs for optimized association analyses, explore the nature of fine-scale recombination and identify regions that may have been subject to natural selection, track the fragments that are transmitted from generation to generation, and gain more insight into the organization of the soybean genome.

Knowledge of the positions of the regions associated with domestication and selection imposed in N. Am. soybean breeding programs is critical for the identification of genes controlling important agronomic traits. Previously, we reported regions related to the domestication and breeding improvement in N. Am. breeding programs using populations with 96 *G. soja*, 96 landraces and 96 elite cultivars (Song *et al.* 2013). However, because of the limited sample size, the span of the regions identified was still quite large. Now with the analysis of a vastly larger number of genotypes, the regions associated with domestication and intensive breeding were identified more narrowly and accurately. We can anticipate that the identification of such regions with strong signatures of selection will be the targets of investigation with the goal of identifying the impacted phenotype and the specific gene/genes that underlie the phenotypic changes.

Using the genotyping data, we identified major candidate regions on seven chromosomes that were significantly associated with seed weight. This is the first report that has narrowly delimited the regions controlling this important trait related to seed yield. Clearly, the candidate genes identified in these regions are of interest for further investigation.

## 

## Supplementary Material

Supporting Information
