# Supplementary material for: Fingerprinting Soybean Germplasm and Its Utility in Genomic Research
Source: G3 (Bethesda). 2015 Jul 28;5(10):1999–2006. doi: 10.1534/g3.115.019000 (PMC4592982; doi:10.1534/g3.115.019000)
Supplement: Supporting Information [file supp_5_10_1999__index.html]

Fingerprinting Soybean Germplasm and Its Utility in Genomic Research — Supporting Information 

# Fingerprinting Soybean Germplasm and Its Utility in Genomic Research

## Supporting Information for Song *et al.*, 2015

**Files in this Data Supplement:**

- Supporting Information - Figure S1 and Tables S1-S11 (PDF, 514 KB)
- Figure S1 - Significance level of genome-wide association of seed weight with SNP loci. (PDF, 445 KB)
- Table S5 - Haplotype block sharing among wild, landrace and North American cultivar populations. (PDF, 84 KB)
- Table S6 - The distribution of haplotype block numbers and their frequencies in the euchromatic and heterochromatic regions of the wild, landrace and North American cultivar populations. (PDF, 95 KB)
- Table S7 - Observed and expected number of genes in the haplotype blocks of euchromatic and heterochromatic regions based on the assumption of 38,381 genes in euchromatic and 8,059 genes in heterochromatic regions. (PDF, 85 KB)
- Table S8 - Observed and expected total recombination rate in the haplotype blocks of euchromatic and heterochromatic regions based on the genetic linkage map length of the Williams 82 ×PI479752 RIL population. (PDF, 85 KB)
- Table S10 - Average Fst and proportion of loci with Fst significant at the 5% probability level in wild vs. landrace and in landrace vs. North American cultivar populations in the 20 chromosomes. (PDF, 86 KB)
- Table S11 - SNPs significantly associated with seed weight and the seed weight QTL previously reported in similar regions based upon QTL analysis. (PDF, 90 KB)
- Table S1 - Description of 19,648 *Glycine max* and *G. soja* accessions genotyped with the SoySNP50K BeadChip. (.xlsx, 810 KB)
- Table S2 - Positions of haplotype blocks in the 806 wild soybean (*G. soja*) accessions. (.xlsx, 220 KB)
- Table S3 - Positions of haplotype blocks in the 5,396 landrace accessions. (.xlsx, 252 KB)
- Table S4 - Positions of haplotype blocks in the 562 North American cultivars. (.xlsx, 157 KB)
- Table S9 - FST of Wild versus landrace populations and landrace versus North American cultivar populations at 42,449 SNP loci across the 20 soybean chromosomes. (.xlsx, 2 MB)
