# Supplementary material for: Fingerprinting Soybean Germplasm and Its Utility in Genomic Research
Source: G3 (Bethesda). 2015 Jul 28;5(10):1999–2006. doi: 10.1534/g3.115.019000 (PMC4592982; doi:10.1534/g3.115.019000)
Supplement: Supporting Information [file supp_g3.115.019000_019000SI.pdf]

G3 · Genes | Genomes | Genetics

Fingerprinting soybean germplasm and its utility in genomic research

Qijian Song[*], David L. Hyten[*1], Gaofeng Jia*, Charles V. Quigley*, Edward W. Fickus[*],

Randall L. Nelson[§] and Perry B. Cregan[*]

[*] United States Department of Agriculture, Agricultural Research Service, Soybean Genomics and Improvement Lab., Beltsville, Maryland, USA

[§] United States Department of Agriculture, Agricultural Research Service, Soybean/Maize Germplasm, Pathology and Genetics Research Unit and Department of Crop Sciences, University of Illinois, Urbana, Illinois, USA

[1] Pioneer Hi-Bred International Inc, Johnston, Iowa, USA.

URL. The dataset for 19,648 soybean Plant Introductions genotyped with the SoySNP50K BeadChip is available at SoyBase, the USDA-ARS Soybean Genetics and Genomics Database (http://soybase.org/snps/download.php).

Correspondence author: Qijian Song, Soybean Genomics and Improvement Laboratory, Beltsville Agricultural Research Center – West, USDA, ARS, Beltsville, MD 20705-2350. Tel: 301-504-5723, e-mail: Qijian.Song@ars.usda.gov

DOI: 10.1534/g3.115.019000

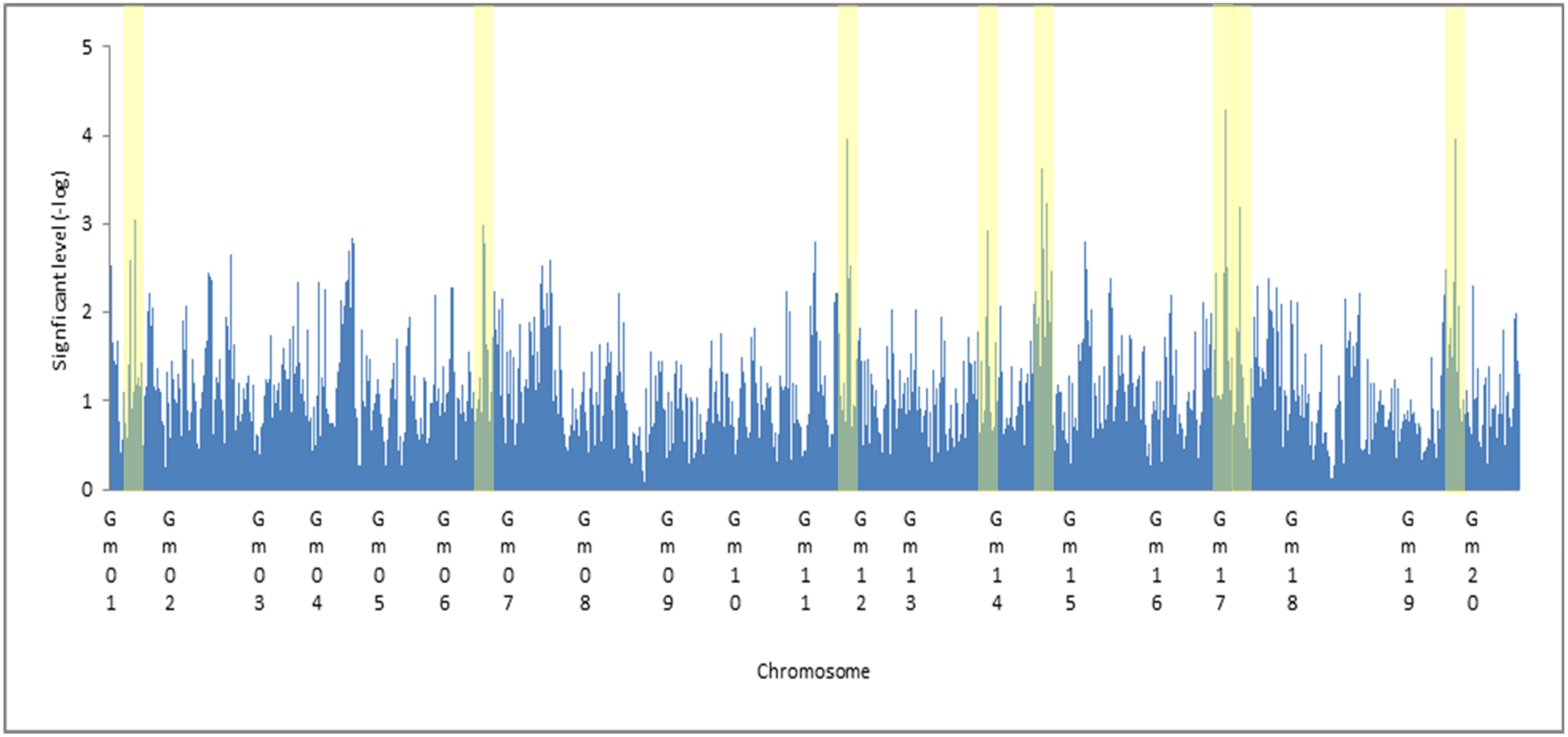

Figure S1 Significance level of genome-wide association of seed weight with SNP loci. Manhatten plot of the negative logarithm of the association P values of SNPs across the 20 soybean chromosomes (Gm01-Gm20) with soybean seed size. The genome positions of eight significant associations are highlighted in yellow.

Tables S1-S4

Available for download as Excel files at www.g3journal.org/lookup/suppl/doi:10.1534/g3.115.019000/-/DC1

Table S1 Description of 19,648 *Glycine max* and *G. soja* accessions genotyped with the SoySNP50K BeadChip. Data include the PI (Plant Introduction) number, Species (*G. max* vs. *G. soja*), Country of origin, Cultivar name if applicable, Maturity Group, 99.9% similar to another accession (Y/N), Landrace or North American Elite cultivars used for analysis and seed weight of accessions used in the association analysis of seed weight.

Table S2 Positions of haplotype blocks in the 806 wild soybean (*G. soja*) accessions. Data include the chromosome, the bp start and end positions of the haplotype block, the size in kbp of the haplotype block and the number of SNPs in the haplotype block based on the Glyma 1.01 genome assembly.

Table S3 Positions of haplotype blocks in the 5,396 landrace accessions. Data include the chromosome, the bp start and end positions of the haplotype block, the size in kbp of the haplotype block and the number of SNPs in the haplotype block based on the Glyma1.01 genome assembly.

Table S4 Positions of haplotype blocks in the 562 North American cultivars. Data include the chromosome, the bp start and end positions of the haplotype block, the size in kbp of the haplotype block, the number of SNPs in the haplotype block based on the Glyma1.01 genome assembly.

Table S5 Haplotype block sharing among wild, landrace and North American cultivar populations

	Number of common SNPs in pair-wise population comparisons	Non-concordant pairs	Concordant pairs	Haplotype block sharing (%)
Wild vs. landrace	11692	2028	17879	89.8
Wild vs. N. Am.	10101	1666	15955	90.5
Landrace vs. N. Am.	21081	13121	67391	83.7

Table S6 The distribution of haplotype block numbers and their frequencies in the euchromatic and heterochromatic regions of the wild, landrace and North American cultivar populations

Frequency	Euchromatic regions			Heterochromatic Regions		
	Wild(%)	Landrace (%)	N. Am.(%)	Wild(%)	Landrace (%)	N. Am.(%)
≤0.1	3412 (23.8)	5850(33.2)	3177(32.6)	681(44.4)	825(43.1)	354(30.0)
>0.1 and ≤0.2	2941(20.5)	3242(18.4)	1508(15.5)	314(20.5)	369(19.3)	184(15.6)
>0.2 and ≤0.3	2116(14.7)	2121(12.0)	1287(13.2)	193(12.6)	211(11.0)	150(12.7)
>0.3 and ≤0.4	1471(10.2)	1694(9.6)	957(9.8)	144(9.4)	102(5.3)	167(14.2)
>0.4 and ≤0.5	1280(8.9)	1392(7.9)	793(8.1)	74(4.8)	71(3.7)	98(8.3)
>0.5 and ≤0.6	1084(7.6)	1129(6.4)	664(6.8)	68(4.4)	95(5.0)	98(8.3)
>0.6 and ≤0.7	894(6.2)	845(4.8)	553(5.7)	37(2.4)	78(4.1)	70(5.9)
>0.7 and ≤0.8	716(5.0)	670(3.8)	434(4.4)	14(0.9)	55(2.9)	34(2.9)
>0.8 and ≤0.9	336(2.3)	522(3.0)	314(3.2)	8(0.5)	89(4.7)	17(1.4)
>0.9 and <1.0	106(0.7)	152(0.9)	66(0.7)	1(0.1)	17(0.9)	8(0.7)

Table S7 Observed and expected number of genes in the haplotype blocks of euchromatic and heterochromatic regions based on the assumption of 38,381 genes in euchromatic and 8,059 genes in heterochromatic regions

Population	Euchromatic regions in haplotype blocks		Heterochromatic regions in haplotype blocks	
	Observed number of genes	Expected number of genes based on proportion of euchromatic regions in blocks genome-wide	Observed number of genes	Expected number of genes based on proportion of heterochromatic regions in blocks genome-wide
Wild	3587	3818	1496	2498
Landrace	16294	15654	4082	4594
N. Am.	18629	18327	3397	3627
Genome-wide	38381		8059	

Table S8 Observed and expected total recombination rate in the haplotype blocks of euchromatic and heterochromatic regions based on the genetic linkage map length of the Williams 82 ×PI479752 RIL population

Population	Total genetic distance in haplotype blocks (cM)	Euchromatic Regions			Heterochromatic Regions		
		Recombination rate in haplotype blocks (cM/Mb)	Expected recombination rate (cM/Mb)	Observed vs. expected total recombination rate (%)	Recombination rate in the haplotype blocks (cM/Mb)	Expected recombination rate (cM/Mb)	Observed vs. expected total recombination rate (%)
Wild	34.6	0.60	4.86	12.3	0.05	0.37	12.4
Landrace	377.9	1.81	4.86	37.3	0.13	0.37	34.1
N. Am. cultivar	653.3	1.90	4.86	39.1	0.15	0.37	41.3

Table S9 F_{ST} of Wild versus landrace populations and landrace versus North American cultivar populations at 42,449 SNP loci across the 20 soybean chromosomes

Available for download as an Excel file at www.g3journal.org/lookup/suppl/doi:10.1534/g3.115.019000/-/DC1

Table S10 Average F_{st} and proportion of loci with Fst significant at the 5% probability level in wild vs. landrace and in landrace vs. North American cultivar populations in the 20 chromosomes

Chr	F_{st} wild vs. landrace	Standard deviation of F_{st} wild vs. landrace	F_{st} landrace vs. N. Am.	Standard deviation of F_{st} landrace vs. N. Am.	Proportion of loci with F_{st} significant at the 5% level wild vs. landrace (%)	Proportion of loci with F_{st} significant at the 5% level landrace vs. N. Am. (%)
Gm01	0.257	0.252	0.100	0.140	7.79	5.25
Gm02	0.229	0.229	0.079	0.116	5.36	3.86
Gm03	0.194	0.229	0.106	0.131	5.51	4.74
Gm04	0.225	0.233	0.115	0.186	4.98	8.52
Gm05	0.261	0.278	0.060	0.096	10.54	2.21
Gm06	0.221	0.234	0.151	0.192	4.96	13.09
Gm07	0.224	0.239	0.074	0.107	6.49	1.74
Gm08	0.248	0.245	0.119	0.157	6.53	9.40
Gm09	0.213	0.222	0.097	0.132	4.43	4.05
Gm10	0.252	0.251	0.111	0.147	7.10	6.20
Gm11	0.274	0.256	0.089	0.131	8.93	4.33
Gm12	0.315	0.291	0.127	0.187	14.05	10.82
Gm13	0.224	0.244	0.126	0.171	6.70	9.93
Gm14	0.232	0.239	0.086	0.106	6.87	1.93
Gm15	0.211	0.218	0.080	0.101	3.41	2.63
Gm16	0.171	0.202	0.140	0.178	3.18	12.48
Gm17	0.203	0.221	0.095	0.119	3.99	3.84
Gm18	0.174	0.189	0.075	0.121	1.59	3.54
Gm19	0.269	0.259	0.157	0.170	8.85	11.86
Gm20	0.257	0.272	0.098	0.142	10.32	4.71
**Mean**	**0.230**	**0.242**	**0.110**	**0.147**	**6.579**	**6.257**

Table S11 SNPs significantly associated with seed weight and the seed weight QTL previously reported in similar regions based upon QTL analysis

Chromosome	Position (bp)	SNP	Significance level (-log(p))	Linkage Group	Previously reported QTL *	Markers associated with previously reported QTL
1	32259879	Gm01_32259879_A_G	3.1	D1a		
6	19735501	Gm06_19735501_A_G	3.0	C2	Sd wt 2-2<br>Sd wt 6-5<br>Sd wt 15-1	A635_1<br>Satt277<br>Satt277
11	28275032	Gm11_28275032_T_C	3.7	B1	Sd wt 10-3	Sat_095
11	28383645	Gm11_28383645_A_G	3.4	B1		
11	28631552	Gm11_28631552_A_G	3.8	B1		
11	30066578	Gm11_30066578_A_G	4.0	B1		
13	38442998	Gm13_38442998_C_T	2.9	F	Sd wt 35-10	Satt502-Satt383
14	28503986	Gm14_28503986_G_T	3.4	B2	Sd wt 10-4<br>Sd wt 13-3	BLT057_2<br>Satt070
14	28527246	Gm14_28527246_T_C	3.6	B2		
14	28574821	Gm14_28574821_A_G	3.6	B2		
14	29397454	Gm14_29397454_A_C	3.0	B2		
14	34265654	Gm14_34265654_T_C	3.2	B2		
17	2415553	Gm17_2415553_G_A	3.1	D2	Sd wt 3-1<br>Sd wt 13-5<br>Sd wt 14-1<br>Sd wt 18-2<br>Sd wt 21-2<br>Sd wt 22-3	A257_1<br>Satt154<br>Satt014<br>Satt002<br>B146_1<br>B146_1
17	2420297	Gm17_2420297_T_C	3.4	D2		
17	2425781	Gm17_2425781_T_C	3.5	D2		
17	2429917	Gm17_2429917_T_G	3.6	D2		
17	2431342	Gm17_2431342_T_G	3.4	D2		
17	2437182	Gm17_2437182_A_C	3.5	D2		
17	2437767	Gm17_2437767_C_A	3.4	D2		
17	2444576	Gm17_2444576_A_G	3.2	D2		
17	2500016	Gm17_2500016_T_C	4.3	D2		
17	2500333	Gm17_2500333_T_G	4.2	D2		
17	8735403	Gm17_8735403_G_A	3.2	D2		
17	8818072	Gm17_8818072_A_C	3.1	D2		
17	8827651	Gm17_8827651_C_T	3.1	D2		
19	42791352	Gm19_42791352_T_C	3.0	L	Sd wt 5-1<br>Sd wt 7-7<br>Sd wt 15-7<br>Sd wt 17-1	Satt527<br>Sat_099<br>Satt113<br>Satt156
19	42812863	Gm19_42812863_T_C	3.5	L		
19	43013222	Gm19_43013222_C_A	4.0	L		
19	43030068	Gm19_43030068_A_G	3.9	L		
19	43117852	Gm19_43117852_A_C	3.8	L		

* QTL names are those assigned by SoyBase (http://www.soybase.org/)

Sd wt 2-2/Sd wt 3-1: Mian et al. 1996. Theor. Appl. Genet 93:1011-1016.
Sd wt 5-1/Sd wt 6-5/Sd wt7-7: Orf et al. 1999. Crop Sci. 39(6):1624-1651.
Sd wt 10-3/Sd wt 10-4: Specht et al. 2001. Crop Sci. 41(2):493-509

Sd wt 13-3/Sd wt 13-5: Hoeck et al. 2003. Crop Sci. 43(1):68-74
Sd wt 14-1: Chapman et al. 2003 Euphytica 129(3):387-393
Sd wt 15-1/Sd wt 15-7: Hyten et al. 2004.Theor. Appl. Genet. 109(3):552-561
Sd wt 17-1: Stombaugh et al. 2004 Crop Sci. 44:2101-2106
Sd wt 18-2: Panthee et al. 2005 Crop Sci. 45(5):2015-2022
Sd wt 21-2: Gai et al. 2007 Front. of Ag. in China 1(1):1-7
Sd wt 22-3: Zhang et al, 2004 Theor. Appl. Genet. 108:1131-1139
Sd wt 35-10: Han et al. 2012 Theor. Appl. Genet. 125 (4):671-683.
