# Supplementary material for: Fingerprinting Soybean Germplasm and Its Utility in Genomic Research
Source: G3 (Bethesda). 2015 Jul 28;5(10):1999–2006. doi: 10.1534/g3.115.019000 (PMC4592982; doi:10.1534/g3.115.019000)
Supplement: Supporting Information [file supp_g3.115.019000_FigureS1.pdf]

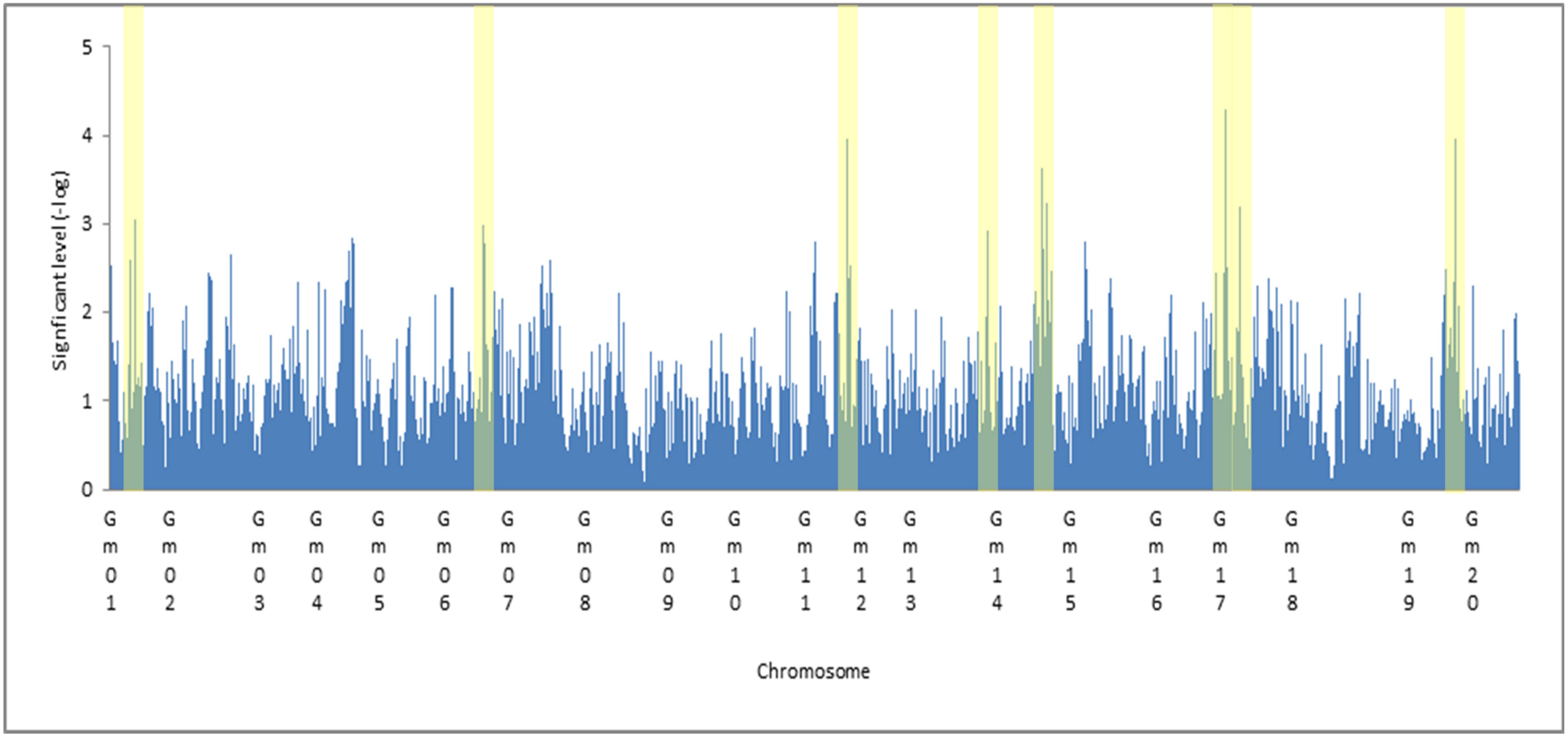

Figure S1 Significance level of genome-wide association of seed weight with SNP loci. Manhatten plot of the negative logarithm of the association P values of SNPs across the 20 soybean chromosomes (Gm01-Gm20) with soybean seed size. The genome positions of eight significant associations are highlighted in yellow.
