# Supplementary material for: Fingerprinting Soybean Germplasm and Its Utility in Genomic Research
Source: G3 (Bethesda). 2015 Jul 28;5(10):1999–2006. doi: 10.1534/g3.115.019000 (PMC4592982; doi:10.1534/g3.115.019000)
Supplement: Supporting Information [file supp_g3.115.019000_TableS5.pdf]

Table S5 Haplotype block sharing among wild, landrace and North American cultivar populations

	Number of common SNPs in pair-wise population comparisons	Non-concordant pairs	Concordant pairs	Haplotype block sharing (%)
Wild vs. landrace	11692	2028	17879	89.8
Wild vs. N. Am.	10101	1666	15955	90.5
Landrace vs. N. Am.	21081	13121	67391	83.7
