# Supplementary material for: Fingerprinting Soybean Germplasm and Its Utility in Genomic Research
Source: G3 (Bethesda). 2015 Jul 28;5(10):1999–2006. doi: 10.1534/g3.115.019000 (PMC4592982; doi:10.1534/g3.115.019000)
Supplement: Supporting Information [file supp_g3.115.019000_TableS6.pdf]

Table S6 The distribution of haplotype block numbers and their frequencies in the euchromatic and heterochromatic regions of the wild, landrace and North American cultivar populations

	Euchromatic regions			Heterochromatic Regions		
Frequency	Wild(%)	Landrace (%)	N. Am.(%)	Wild(%)	Landrace (%)	N. Am.(%)
≤0.1	3412 (23.8)	5850(33.2)	3177(32.6)	681(44.4)	825(43.1)	354(30.0)
>0.1 and ≤0.2	2941(20.5)	3242(18.4)	1508(15.5)	314(20.5)	369(19.3)	184(15.6)
>0.2 and ≤0.3	2116(14.7)	2121(12.0)	1287(13.2)	193(12.6)	211(11.0)	150(12.7)
>0.3 and ≤0.4	1471(10.2)	1694(9.6)	957(9.8)	144(9.4)	102(5.3)	167(14.2)
>0.4 and ≤0.5	1280(8.9)	1392(7.9)	793(8.1)	74(4.8)	71(3.7)	98(8.3)
>0.5 and ≤0.6	1084(7.6)	1129(6.4)	664(6.8)	68(4.4)	95(5.0)	98(8.3)
>0.6 and ≤0.7	894(6.2)	845(4.8)	553(5.7)	37(2.4)	78(4.1)	70(5.9)
>0.7 and ≤0.8	716(5.0)	670(3.8)	434(4.4)	14(0.9)	55(2.9)	34(2.9)
>0.8 and ≤0.9	336(2.3)	522(3.0)	314(3.2)	8(0.5)	89(4.7)	17(1.4)
>0.9 and <1.0	106(0.7)	152(0.9)	66(0.7)	1(0.1)	17(0.9)	8(0.7)
