# Supplementary material for: Fingerprinting Soybean Germplasm and Its Utility in Genomic Research
Source: G3 (Bethesda). 2015 Jul 28;5(10):1999–2006. doi: 10.1534/g3.115.019000 (PMC4592982; doi:10.1534/g3.115.019000)
Supplement: Supporting Information [file supp_g3.115.019000_TableS7.pdf]

Table S7 Observed and expected number of genes in the haplotype blocks of euchromatic and heterochromatic regions based on the assumption of 38,381 genes in euchromatic and 8,059 genes in heterochromatic regions

Population	Euchromatic regions in haplotype blocks		Heterochromatic regions in haplotype blocks	
	Observed number of genes	Expected number of genes based on proportion of euchromatic regions in blocks genome-wide	Observed number of genes	Expected number of genes based on proportion of heterochromatic regions in blocks genome-wide
Wild	3587	3818	1496	2498
Landrace	16294	15654	4082	4594
N. Am.	18629	18327	3397	3627
Genome-wide	38381		8059	
