# Supplementary material for: Fingerprinting Soybean Germplasm and Its Utility in Genomic Research
Source: G3 (Bethesda). 2015 Jul 28;5(10):1999–2006. doi: 10.1534/g3.115.019000 (PMC4592982; doi:10.1534/g3.115.019000)
Supplement: Supporting Information [file supp_g3.115.019000_TableS8.pdf]

Table S8 Observed and expected total recombination rate in the haplotype blocks of euchromatic and heterochromatic regions based on the genetic linkage map length of the Williams 82 ×PI479752 RIL population

Population	Total genetic distance in haplotype blocks (cM)	Euchromatic Regions			Heterochromatic Regions		
		Recombination rate in haplotype blocks (cM/Mb)	Expected recombination rate (cM/Mb)	Observed vs. expected total recombination rate (%)	Recombination rate in the haplotype blocks (cM/Mb)	Expected recombination rate (cM/Mb)	Observed vs. expected total recombination rate (%)
Wild	34.6	0.60	4.86	12.3	0.05	0.37	12.4
Landrace	377.9	1.81	4.86	37.3	0.13	0.37	34.1
N. Am. cultivar	653.3	1.90	4.86	39.1	0.15	0.37	41.3
