# Supplementary material for: Fingerprinting Soybean Germplasm and Its Utility in Genomic Research
Source: G3 (Bethesda). 2015 Jul 28;5(10):1999–2006. doi: 10.1534/g3.115.019000 (PMC4592982; doi:10.1534/g3.115.019000)
Supplement: Supporting Information [file supp_g3.115.019000_TableS10.pdf]

Table S10 Average F_{st} and proportion of loci with Fst significant at the 5% probability level in wild vs. landrace and in landrace vs. North American cultivar populations in the 20 chromosomes

Chr	F_{st} wild vs. landrace	Standard deviation of F_{st} wild vs. landrace	F_{st} landrace vs. N. Am.	Standard deviation of F_{st} landrace vs. N. Am.	Proportion of loci with F_{st} significant at the 5% level wild vs. landrace (%)	Proportion of loci with F_{st} significant at the 5% level landrace vs. N. Am. (%)
Gm01	0.257	0.252	0.100	0.140	7.79	5.25
Gm02	0.229	0.229	0.079	0.116	5.36	3.86
Gm03	0.194	0.229	0.106	0.131	5.51	4.74
Gm04	0.225	0.233	0.115	0.186	4.98	8.52
Gm05	0.261	0.278	0.060	0.096	10.54	2.21
Gm06	0.221	0.234	0.151	0.192	4.96	13.09
Gm07	0.224	0.239	0.074	0.107	6.49	1.74
Gm08	0.248	0.245	0.119	0.157	6.53	9.40
Gm09	0.213	0.222	0.097	0.132	4.43	4.05
Gm10	0.252	0.251	0.111	0.147	7.10	6.20
Gm11	0.274	0.256	0.089	0.131	8.93	4.33
Gm12	0.315	0.291	0.127	0.187	14.05	10.82
Gm13	0.224	0.244	0.126	0.171	6.70	9.93
Gm14	0.232	0.239	0.086	0.106	6.87	1.93
Gm15	0.211	0.218	0.080	0.101	3.41	2.63
Gm16	0.171	0.202	0.140	0.178	3.18	12.48
Gm17	0.203	0.221	0.095	0.119	3.99	3.84
Gm18	0.174	0.189	0.075	0.121	1.59	3.54
Gm19	0.269	0.259	0.157	0.170	8.85	11.86
Gm20	0.257	0.272	0.098	0.142	10.32	4.71
**Mean**	**0.230**	**0.242**	**0.110**	**0.147**	**6.579**	**6.257**
