# Supplementary material for: Fingerprinting Soybean Germplasm and Its Utility in Genomic Research
Source: G3 (Bethesda). 2015 Jul 28;5(10):1999–2006. doi: 10.1534/g3.115.019000 (PMC4592982; doi:10.1534/g3.115.019000)
Supplement: Supporting Information [file supp_g3.115.019000_TableS11.pdf]

Table S11 SNPs significantly associated with seed weight and the seed weight QTL previously reported in similar regions based upon QTL analysis

| Chromosome | Position (bp) | SNP | Significance level (-log(p)) | Linkage Group | Previously reported QTL * | Markers associated with previously reported QTL |
|---|---|---|---|---|---|---|
| 1 | 32259879 | Gm01_32259879_A_G | 3.1 | D1a | | |
| 6 | 19735501 | Gm06_19735501_A_G | 3.0 | C2 | Sd wt 2-2<br>Sd wt 6-5<br>Sd wt 15-1 | A635_1<br>Satt277<br>Satt277 |
| 11 | 28275032 | Gm11_28275032_T_C | 3.7 | B1 | Sd wt 10-3 | Sat_095 |
| 11 | 28383645 | Gm11_28383645_A_G | 3.4 | B1 | | |
| 11 | 28631552 | Gm11_28631552_A_G | 3.8 | B1 | | |
| 11 | 30066578 | Gm11_30066578_A_G | 4.0 | B1 | | |
| 13 | 38442998 | Gm13_38442998_C_T | 2.9 | F | Sd wt 35-10 | Satt502-Satt383 |
| 14 | 28503986 | Gm14_28503986_G_T | 3.4 | B2 | Sd wt 10-4<br>Sd wt 13-3 | BLT057_2<br>Satt070 |
| 14 | 28527246 | Gm14_28527246_T_C | 3.6 | B2 | | |
| 14 | 28574821 | Gm14_28574821_A_G | 3.6 | B2 | | |
| 14 | 29397454 | Gm14_29397454_A_C | 3.0 | B2 | | |
| 14 | 34265654 | Gm14_34265654_T_C | 3.2 | B2 | | |
| 17 | 2415553 | Gm17_2415553_G_A | 3.1 | D2 | Sd wt 3-1<br>Sd wt 13-5<br>Sd wt 14-1<br>Sd wt 18-2<br>Sd wt 21-2<br>Sd wt 22-3 | A257_1<br>Satt154<br>Satt014<br>Satt002<br>B146_1<br>B146_1 |
| 17 | 2420297 | Gm17_2420297_T_C | 3.4 | D2 | | |
| 17 | 2425781 | Gm17_2425781_T_C | 3.5 | D2 | | |
| 17 | 2429917 | Gm17_2429917_T_G | 3.6 | D2 | | |
| 17 | 2431342 | Gm17_2431342_T_G | 3.4 | D2 | | |
| 17 | 2437182 | Gm17_2437182_A_C | 3.5 | D2 | | |
| 17 | 2437767 | Gm17_2437767_C_A | 3.4 | D2 | | |
| 17 | 2444576 | Gm17_2444576_A_G | 3.2 | D2 | | |
| 17 | 2500016 | Gm17_2500016_T_C | 4.3 | D2 | | |
| 17 | 2500333 | Gm17_2500333_T_G | 4.2 | D2 | | |
| 17 | 8735403 | Gm17_8735403_G_A | 3.2 | D2 | | |
| 17 | 8818072 | Gm17_8818072_A_C | 3.1 | D2 | | |
| 17 | 8827651 | Gm17_8827651_C_T | 3.1 | D2 | | |
| 19 | 42791352 | Gm19_42791352_T_C | 3.0 | L | Sd wt 5-1<br>Sd wt 7-7<br>Sd wt 15-7<br>Sd wt 17-1 | Satt527<br>Sat_099<br>Satt113<br>Satt156 |
| 19 | 42812863 | Gm19_42812863_T_C | 3.5 | L | | |
| 19 | 43013222 | Gm19_43013222_C_A | 4.0 | L | | |
| 19 | 43030068 | Gm19_43030068_A_G | 3.9 | L | | |
| 19 | 43117852 | Gm19_43117852_A_C | 3.8 | L | | |

* QTL names are those assigned by SoyBase (http://www.soybase.org/)

Sd wt 2-2/Sd wt 3-1: Mian et al. 1996. Theor. Appl. Genet 93:1011-1016.
Sd wt 5-1/Sd wt 6-5/Sd wt7-7: Orf et al. 1999. Crop Sci. 39(6):1624-1651.
Sd wt 10-3/Sd wt 10-4: Specht et al. 2001. Crop Sci. 41(2):493-509

Sd wt 13-3/Sd wt 13-5: Hoeck et al. 2003. Crop Sci. 43(1):68-74
Sd wt 14-1: Chapman et al. 2003 Euphytica 129(3):387-393
Sd wt 15-1/Sd wt 15-7: Hyten et al. 2004.Theor. Appl. Genet. 109(3):552-561
Sd wt 17-1: Stombaugh et al. 2004 Crop Sci. 44:2101-2106
Sd wt 18-2: Panthee et al. 2005 Crop Sci. 45(5):2015-2022
Sd wt 21-2: Gai et al. 2007 Front. of Ag. in China 1(1):1-7
Sd wt 22-3: Zhang et al, 2004 Theor. Appl. Genet. 108:1131-1139
Sd wt 35-10: Han et al. 2012 Theor. Appl. Genet. 125 (4):671-683.
